# Associations between dietary total antioxidant capacity and odds of non-alcoholic fatty liver disease (NAFLD) in adults: a case–control study

**DOI:** 10.1017/jns.2020.39

**Published:** 2020-11-11

**Authors:** Mohammad Hassan Sohouli, Somaye Fatahi, Aliakbar Sayyari, Beheshteh Olang, Farzad Shidfar

**Affiliations:** 1 Department of Nutrition, School of Public Health, Iran University of Medical Sciences, Tehran, 1449614535, Iran; 2 Student Research Committee, Faculty of Public Health Branch, Iran University of Medical Sciences, Tehran, 1449614535, Iran; 3 Pediatric Gastroenterology, Hepatology, and Nutrition Research Center, Research Institute for Children's Health, Shahid Beheshti University of Medical Sciences, Tehran, 1546815514, Iran

**Keywords:** Dietary total antioxidant capacity, Obesity, Liver enzyme, Non-alcoholic fatty liver disease

## Abstract

The relationships between the total antioxidant capacity (TAC) of the diet and the risk of non-alcoholic fatty liver disease (NAFLD) have not previously been assessed. The aim of this study was to assess relationships between DTAC and odds of NAFLD in a case–control study. This case–control study was carried out in 158 patients with NAFLD and 357 healthy individuals aged 18–55 years. Dietary data were collected using validated 168-item quantitative food frequency questionnaires. Triacylglycerols (TAGs), total cholesterol (TC), high-density lipoprotein (HDL-C), low-density lipoprotein (LDL-C) and fasting blood glucose (FBS) concentrations were assessed using enzymatic methods and commercial kits. The DTAC was calculated based on the oxygen radical absorbance capacity of each food reported by the U.S. Department of Agriculture. The mean ± sd (standard deviation) for age and body mass index (BMI) of the study participants were 43⋅9 years ±5⋅9 and had 30⋅5 kg/m^2^ ±2⋅6. The NAFLD patients included higher BMI and female proportion, compared with the control group. The NAFLD patients included higher smoking rates, biochemical parameters (TG, TC, LDL-C and FBS) and DTAC scores, compared with control groups (*P*-value < 0⋅05). However, patients with NAFLD had lower HDL levels and physical activities, compared with the control group. The highest tertile of DTAC showed lower odds of NAFLD, compared with the lowest tertile. This association was significant after adjustment for potential confounders (OR, 0⋅19; 95 % CI, 0⋅9–0⋅34; *P* for trend 0⋅001). Findings suggest that the promotion of naturally increased antioxidant capacities may help prevent odds of NAFLD.

## Introduction

Non-alcoholic fatty liver disease (NAFLD) is one of the major global health challenges, which comprises a wide pathological spectrum ranging from simple steatosis to steatohepatitis with variable degrees of fibrosis and cirrhosis^(1,2)^. This disease is a highly prevalent condition characterised by fatty infiltration of liver cells resembling that of alcohol-induced liver injury but occurring in patients who do not abuse alcohol^(3)^. Worldwide, the estimated prevalence rate of NAFLD in general populations is nearly 25 %^(4)^. In Iran, the prevalence rate of NAFLD in children and adults has been reported as 7 and 35 %, respectively^(5)^. Studies have suggested that systemic inflammations or excessive levels of free radicals and disruption of antioxidant balances in the body (generally oxidative stress) may play key roles in pathogenesis of NAFLD through increased lipid peroxidation in cell membranes^(6,7)^. When antioxidant and anti-inflammatory defences are exhausted, chronic states of liver diseases increase. Various aspects of the effects of natural antioxidants in foods on the modulation of oxidative stresses and disorders in antioxidant systems have been studied^(8,9)^. One of the diet components that can affect these symptoms is the natural antioxidant. Interventional studies using antioxidant supplements (vitamins C and E) have shown that supplementation with antioxidants may include positive effects on these symptoms and diseases^(10)^. However, the assessment of an antioxidant compound alone cannot reflect total antioxidant potency of the diet and reflects the synergistic and potential effects of dietary antioxidant interactions. Thus, the term of dietary total antioxidant capacity (DTAC) has been developed as an appropriate tool to assess effects of dietary antioxidants. This includes a strong correlation with serum TAC^(10)^ and is closely linked to the quality of diets to assess risks of chronic diseases^(11)^.

Several evidences have suggested potential links between DTAC and decreased risks of chronic diseases such as diabetes^(11)^, metabolic and oxidative stress markers^(12)^, ulcerative colitis^(13)^, blood pressure^(14)^ and cardiovascular diseases (CVDs)^(15)^, which share common metabolic parameters with NAFLD. However, to the best of the authors’ knowledge, associations between DTAC and odds of NAFLD have not been investigated. Only a recent cross-sectional study on steatohepatitis patients has declared that a high DTAC score is linked to low hepatic injuries through reducing free radical productions and hence decreasing oxidative stresses^(16)^. Considering lack of convincing evidences on associations of DTAC with liver function, the current study was carried out to investigate possible associations between DTAC and odds of NAFLD in the Iranian population.

## Subjects and methods

### Participants

This hospital-based case–control study was performed in Hazrat Rasoul Hospital, Tehran, Iran, 2018–19. About 158 consecutive patients with NAFL diagnosed by a gastroenterologist were included in the case group; 357 individuals without a history of NAFLD were recruited from the same hospital for the control group. Two dietitians (M.H.S. and S.F.) monitored the sampling of patients. Diagnosis of NAFLD was carried out using chronic elevation of liver enzymes, absence of alcohol consumption, ultrasonography (US) results of the liver showing NAFLD (Grades II and III) and exclusion of other aetiologies of liver diseases. Furthermore, NAFLD was diagnosed using US, based on echogenicity and visualisation of vasculature, parenchyma and diaphragm. These were compared with histological features based on Brunt's Classification^(17)^.Case group patients were newly diagnosed and were not treated before the study. Healthy individuals were considered as a control group based on the laboratory tests and liver US (not suffering from stages of hepatic steatosis). The control group was selected from other patients referred to other wards of the hospital such as ophthalmology, orthopaedics, maxillofacial surgery and ear, nose and throat wards, who were not diagnosed with NAFLD and had no history of alcohol overall or had a history of alcohol consumption less than 10 mg/d in women and less than 20 mg/d in men. Case and control group members were matched regarding age and sex. After entering the study, information on demographic variables was collected by completing general information questionnaires. Data about other variables including demographic features age, sex, family size, disease history as well as smoking status were collected through a general information questionnaire. At the beginning of the study, the levels of liver enzymes were recorded using the patient's existing medical record. In the absence of a record, patients were referred to the hospital for blood liver enzymes through a blood sample after a doctor's appointment on a specific day. Also, liver enzyme levels were assessed after return of the participants to the hospital. For the completion of data on dietary intakes and other information, participants were invited to the research centre on a special date. Participants with a history of certain diseases (diabetes, CVDs, myocardial infarctions/strokes, cancers, viral hepatitis, Wilson's disease, and autoimmune disorders of the liver) were excluded from the list. Pregnant and lactating women and subjects with arbitrary special diets were excluded from the list as well. In this study, nutritionists were used as interviewers. Therefore, all patients responded completely to the survey questions. Furthermore, written informed consents were completed for the participants. To assess physical activity levels of the participants, general practice physical activity questionnaires (GPPAQs) were used. The GPPAQ is a simple questionnaire reflecting personal current physical activities^(18)^. This study was approved by the Research Council and Ethics Committee, Iran University of Medical Sciences, Tehran, Iran (No. IR.IUMS.REC.1397.667).

#### Anthropometric assessment

Anthropometric measurements were carried out by a trained dietitian. Weight was measured with minimum clothes and no shoes using a standard SECA 700 Digital Scale (SECA, Hamburg, Germany) and recorded to the nearest 100 g. Height was measured in a standing relaxed shoulder position with no shoes to the nearest 0⋅5 cm using a mounted tape (SECA Stadiometer, Hamburg, Germany). Body mass index (BMI) was calculated as weight (kg) divided by height in square metres (m^2^).

#### Measurement of biomarkers

After 12 h of fasting, 10 ml of fasting blood was collected between 7 and 10 h from all participants. Blood samples were centrifuged at 500 ***g*** for 10 min at 4°C within 30–45 min of the collection. Sera were stored at −80°C until use. Then, triacylglycerols (TAGs), total cholesterol (TC), HDL-C, LDL-C and fasting blood glucose (FBS) concentrations were assessed using enzymatic methods and commercial kits (Pars Azmoon, Tehran, Iran), Noor Laboratory, Iran University of Medical Sciences, Tehran, Iran. Alanine aminotransferase (ALT) and aspartate aminotransferase (AST) were assessed using commercially available enzymatic reagents (Pars Azmoon, Tehran, Iran) and automatic analyzer (BT-3000).

### Dietary assessment and DTAC calculation

Trained dietitians administered usual food intakes during interviews. Validated semi-quantitative food frequency questionnaires (FFQs) with 168 food items were used to assess dietary intakes^(19)^. The validity and reliability of the questionnaire were evaluated in previous studies^(19,20)^. For each food item, a standard unit or portion size was specified and participants were asked how often, on average, during the previous year they had consumed that amount. The participants reported the consumption frequency of each food item per day, week, month or year. Responses to the individual food items were converted to average daily intake of each food item. The nutrient and energy content of the foods were analysed using the Nutritionist 4 software (First DatabankInc., Hearst Corp., San Bruno, CA, USA) modified for Iranian foods. Moreover, DTAC was calculated based on the oxygen radical absorbance capacity of each food (except for coffee) reported by the USDA Oxygen Radical Absorbance Capacity (ORAC) database and expressed as mmol of trolox equivalent/100 g of food (mmol TE/100 g)^(21)^. According to studies, the ORAC index has a higher correlation with serum antioxidant levels than FRAP and TEAC indices^(22,23)^. Also, this index includes more food items and nutrients than other available indices due to more accurate evaluation^(24)^. Conversely, other indicators include spices and even cooked and frozen foods^(23,24)^.

### Statistical analysis

Statistical analysis was carried out using the Statistical Package Software for Social Science v.21 (SPSS Inc., Chicago, IL, USA). The Kolmogorov–Smirnov test and the histogram chart were used for testing normality of the data. The independent Student's *t*-test and Mann–Whitney test were used to compare variables with normal and abnormal distributions, respectively. Baseline characteristics and dietary intakes were expressed as mean ± sd (standard deviation) or median (25–75 interquartile range) for quantitative variables and number and percentages for qualitative variables. The sample size was calculated by considering the type 1 error (a) of 0⋅05 and type 2 error (b) of 0⋅20 (power = 80 %), that primary end point of our study was based on the differences between patients and controls in ORAC. Comparison of data between the two groups was carried out using independent sample *t*-test and *χ*^2^ for continuous and categorical variables, respectively. Using analysis of covariance (ANCOVA), differences of nutrient intakes were compared within DTAC tertiles. A *χ*^2^ test was used for qualitative variables to identify significant differences within quartile categories of DTAC. All confounders (total fat, dietary protein, calcium and CHO) were selected based on the following two strategies of (1) similar literatures, which assessed dietary TAC such as a study by Sotoudeh *et al.*^(25)^ and Rahmani *et al.*^(26)^ that adjusted this index for fat and protein intake; (2) confounders of dietary fat, CHO, protein and calcium differed between the case and control groups (see Table 2) and included significant associations with exposure (DTAC). In general, no studies are available to suggest that these food groups (fat, CHO, protein and calcium) are directly involved in the calculation of DTAC. Binary logistic regression was used to estimate odds ratios (ORs) with 95 % confidence intervals (CIs) adjusted for multiple covariates in various models. Data were presented as mean ± sd and odds ratio with 95 % confidence intervals. Significance levels were reported when *P* < 0⋅05.

## Result

The mean ± sd for age and BMI of the participants were 43⋅9 years ±5⋅9 (females = 58⋅7 %) and 30⋅5 kg/m^2^ ±2⋅6, respectively. Table 1 demonstrates anthropometric and biochemical indices within case (non-alcoholic fatty liver patients) and control groups. No significant differences were seen between the case and control groups in age and sex (*P* 0⋅76) and (*P* 0⋅51), respectively. However, NAFLD patients included higher BMI, compared with the control group (*P* < 0⋅001). Moreover, NAFLD cases include higher levels of smoking, FBS, TG, LDL, TC and ALT, compared with the control group (*P* < 0⋅05). However, patients with NAFLD included lower HDL and DTAC scores as well as physical activities, compared with the control group (*P* < 0⋅05). Dietary intakes in NAFLD case and control groups are presented in Table 2. Significant differences were demonstrated in intakes of more micronutrients (calcium, vitamin E, vitamin B_12_, vitamin D and vitamin C) and macronutrients (protein, fats, saturated fatty acids, carbohydrates, fruits, vegetables, whole grains, refined grains and fructose) between the case and control groups (*P* < 0⋅05). Additionally, no significant differences were found between the case and control groups in intakes of proteins, fibres, selenium and folates. Also, correlations between DTAC and food groups including fruits, vegetables, nuts, legumes, fruit juice, tea and olive oil are presented in Table 3. After adjustment for energy intake and sex, positive correlation was found between DTAC and fruits (*r* 0⋅78; *P* < 0⋅001), vegetables (*r* 0⋅54; *P* < 0⋅001), nuts (*r* 0⋅48; *P* < 0⋅001), legumes (*r* 0⋅33; *P* < 0⋅001), fruit juice (*r* 0⋅33; *P* < 0⋅001), tea (*r* 0⋅25; *P* < 0⋅001) and olive oil (*r* 0⋅18; *P* 0⋅001). Moreover, food groups such as vegetables (31⋅9 %), fruits (29⋅2 %), tea (13 %), legumes (7⋅8 %) and nuts (6⋅7 %) were found to be the main contributors to DTAC. Participants’ characteristics and biochemical parameters within the tertiles of DTAC are shown in Table 4. The mean age significantly increased within the tertiles of DTAC (*P* < 0⋅002), while FBS, TG and ALT significantly decreased within the tertiles of DTAC (*P* < 0⋅05). No significant differences were seen in other characteristics and biochemical parameters and BMI within the tertiles of DTAC. The ORs and 95 % CIs for NAFLD in the tertiles of DTAC are described in Table 5. Compared with participants in the lowest tertile of DTAC, those in the highest tertile included significantly lower ORs for NAFLD (crude model: OR, 0⋅37; 95 % CI, 0⋅24, 0⋅58; *P* for trend <0⋅001), which were still significant after further adjustments for BMI, physical activities and dietary intakes of fats, protein, carbohydrates, calcium and energy as well as biochemical parameters (Model 3: OR, 0⋅19; 95 % CI, 0⋅9, 0⋅34; *P* for trend 0⋅001).

**Table 1.** Anthropometric and biochemical parameters of the case (non-alcoholic fatty liver patients) and control groups

Variable	Group, mean ± sd		*P*-value
	Case (*n* 158)	Control (*n* 357)	
Women, % (no)	46·3 (98)	45·9 (88)	0·51*
Age, years	45·75 ± 5·9	45·13 ± 5·9	0·76**
BMI, kg/m^2^	33·19 ± 3·1	27·95 ± 2·1	<0·001**
Physical activity (Met·min/week)	984·5 ± 1241·5	1563·2 ± 1216·7	<0·001**
Smoking (yes), *n* (%)	16 (7·1)	12 (2·7)	0·006
FBS, mg/dl	109·29 ± 7·6	92·21 ± 6·7	<0·001**
Total cholesterol, mg/dl	184·79 ± 42·1	182·85 ± 40·1	<0·001**
Triacylglycerols, mg/dl	180·40 ± 14·3	130·99 ± 12·2	0·001**
HDL, mg/dl	41·26 ± 15·1	48·5 ± 17·3	0·017**
LDL, mg/dl	121·17 ± 23·4	109·14 ± 13·0	<0·001**
ALT, mg/dl	58·50 ± 24·1	20·53 ± 13·01	<0·001**
AST, mg/dl	34·88 ± 17·2	23·76 ± 9·6	0·17**

BMI, body mass index; FBS, fasting blood sugar; HDL, high-density lipoprotein; LDL, low-density lipoprotein; ALT, alanine aminotransferase; AST, aspartate aminotransferase.

*P-values are resulted from chi-square test.

**P-values are resulted from Student's *t*-test.

**Table 2.** Dietary intakes by the case (non-alcoholic fatty liver patients) and control groups

Variable	Group, mean ± sd		*P*-value*
	Case (*n* 158)	Control (*n* 357)	
Energy, kcal	2741⋅8 ± 819⋅89	2427⋅67 ± 798⋅09	0·04
Macronutrients			
Protein (%)	14·19 ± 3·3	17·21 ± 4·11	<0·001
Fat (%)	33·95 ± 7·82	31·98 ± 6·69	0·01
Carbohydrate (%)	59·53 ± 6·37	62·35 ± 10·12	<0·001
Saturated fatty acid (g/d)	32·13 ± 6·35	26·87 ± 6·42	0·006
Fibre (g/d)	46·36 ± 18·25	47·58 ± 19·76	0·5
Fruits (g/d)	249·38 ± 190·29	368·03 ± 192·15	<0·001
Vegetables (g/d)	269·36 ± 183·63	339·31 ± 185·25	<0·001
Whole grain (g/d)	121·54 ± 80·71	134·44 ± 79·91	<0·001
Refined grain (g/d)	420·41 ± 140·35	368·87 ± 141·65	<0·001
Fructose (g/d)	25·95 ± 12·19	23·52 ± 10·33	0·03
Legumes (g/d)	18·8 ± 1·5	17·4 ± 0·7	0·367
Fish (g/d)	9·3 ± 0·5	9·4 ± 0·4	0·897
Red and organ meats (g/d)	26·1 ± 1·6	22·0 ± 0·9	0·023
Processed meats (g/d)	8·8 ± 9·4	2·6 ± 4·3	<0·001
High-fat dairy (g/d)	129·8 ± 7·6	55·2 ± 3·1	<0·001
Low-fat dairy (g/d)	229·9 ± 11·5	224·2 ± 7·8	0·679
Coffee and tea (g/d)	619·6 ± 38·4	613·6 ± 25·2	0·894
Nuts (g/d)	6·3 ± 0·4	6·3 ± 0·5	0·909
*Micronutrients*			
Calcium (mg/d)	1125·66 ± 381·61	1311·17 ± 441·93	<0·001
Selenium (mg/d*)*	135·39 ± 56·28	133·34 ± 52·41	0·717
Vitamin E (mg/d)	13·22 ± 5·866	18·98 ± 7·146	<0·001
Folate (μg/d)	615·53 ± 182·21	642·29 ± 194·52	0·176
Vitamin B12 (μg/d)	6·37 ± 4·47	5·36 ± 4·42	0·029
Vitamin D (μg/d)	1·56 ± 1·27	2·03 ± 1·80	0·03
Vitamin C (mg/d)	175·72 ± 88·09	231·92 ± 144·06	<0·001
DTAC, mmol TE/100 g	12323·6 ± 5398·5	17563·4 ± 8247·2	<0·001^†^

TE, trolox equivalents; DTAC, dietary total antioxidant capacity.

* *P-*values are resulted from Student's *t*-test.

**Table 3.** Correlations between DTAC and food groups

Variable	Partial correlations	*P*-value
	*r*	
Fruits	0⋅86	<0⋅001
Vegetables	0⋅52	<0⋅001
Nuts	0⋅50	<0⋅001
Legumes	0⋅41	<0⋅001
Fruit juice	0⋅35	<0⋅001
Tea	0⋅19	<0⋅001
Olive oil	0⋅19	0⋅001

DTAC, dietary total antioxidant capacity.

**Table 4.** Characteristics and biochemical parameters within tertiles of dietary total antioxidant capacity in the study population

	Tertiles of dietary total antioxidant capacity			P*
	T1 (*n* 171)	T2 (*n* 173)	T3 (*n* 171)	
Age (year)	37·0 ± 8·3	38·2 ± 8·5	39·5 ± 9·6	0·002
BMI, kg/m^2^	26·8 ± 4·1	26·7 ± 4·4	26·9 ± 4·4	0·687
Physical activity (Met·min/week)	1462 ± 862	1454 ± 850	1368 ± 939	0·261
Smoking (yes), *n* (%)	9 (3·5)	11 (4·9)	8 (4·2)	0·722
FBS, mg/dl	102·73 ± 43·92	95·20 ± 36·79	94·81 ± 33·58	0·001
Total cholesterol, mg/dl	186·34 ± 41·68	182·20 ± 43·70	183·27 ± 46·52	0·804
Triacylglycerols, mg/dl	155·56 ± 114·17	144·46 ± 72·26	141·09 ± 79·87	<0·001
HDL, mg/dl	47·09 ± 12·02	44·34 ± 12·00	47·52 ± 14·61	0·198
LDL, mg/dl	113·42 ± 31·53	111·98 ± 35·75	111·11 ± 35·77	0·261
ALT, mg/dl	39·24 ± 51·22	30·49 ± 25·78	29·78 ± 38·03	<0·001
AST, mg/dl	46·14 ± 63·17	45· 91 ± 62·89	42·36 ± 59·63	0·785

* *P*-value from one-factor ANCOVA test or *χ*^2^ test, for continuous or categorical variables, respectively.

**Table 5.** Associations between tertiles of dietary total antioxidant capacity and odds of NAFLD in participants

	Tertile of dietary total antioxidant capacity			*P* for trend
	T1	T2	T3	
DTAC				
Case/control	61/105	58/125	39/127	
Crude	1⋅00 (Ref.)	0⋅76 (0⋅52–1⋅12)	0⋅37 (0⋅24–0⋅58)	<0⋅001
Model 1^a^	1⋅00 (Ref.)	0⋅74 (0⋅42–1⋅28)	0⋅20 (0⋅10–0⋅40)	<0⋅001
Model 2^b^	1⋅00 (Ref.)	0⋅83 (0⋅33–1⋅64)	0⋅39 (0⋅19–0⋅82)	0⋅008
Model 3^c^	1⋅00 (Ref.)	0⋅62 (0·32–1⋅07)	0⋅19 (0⋅9–0⋅34)	0⋅001

Note: Binary logistic regression was used to estimate odds ratios (ORs) and 95 % confidence intervals (CIs) adjusted for multiple covariates in different models.

DTAC, dietary total antioxidant capacity (DTAC).

a Model 1: adjusted for age and sex.

b Model 2: adjusted for model 1 and BMI, physical activity, dietary intake of fat, protein, carbohydrate, calcium and energy.

c Model 3: additionally adjusted for fasting blood sugar, TG, cholesterol, LDL-C, and HDL-C and smoking at baseline.

## Discussion

The current case–control study investigated associations between DTAC and NAFLD. Generally, DTAC included inverse associations with weight, FBS, TG and ALT. Participants with higher DTAC scores included significantly lower ORs in NAFLD, after adjustment for BMI, physical activity, education level, and dietary intakes of protein, carbohydrate, fats, energy, calcium as well as biochemical parameters, compared with low DTAC scores. In the present study, the case group included lower DTAC scores, compared with the control group, suggesting that DTAC may be a potential predictor of the odds of NAFLD. To the best of the authors’ knowledge, the current study is the first documentation of inverse associations between DTAC and odds of NAFLD. In previous studies, dietary TAC has inversely been associated with several chronic diseases such as CVDs^(15)^, cancers^(27)^, diabetes^(11)^ and metabolic disorders^(12)^, which share common metabolic parameters with NAFLD. Only one case–control study has assessed status of the blood redox with the severity of NAFLD^(28)^ with no differences in dietary antioxidant scores between the two groups of case and control. In the highlighted study, no calculations of the odds or risks of the disease were carried out and the sample size was small. Although, thiobarbituric acid reactive substances were significantly associated with the likelihood of NAFLD, independently diet's total antioxidant.

Overall, inverse associations were reported between intakes of antioxidant micronutrients and antioxidant-rich foods and risks of NAFLD. For example, DASH diets (Dietary Approaches to Stop Hypertension), dietary patterns with antioxidant-rich components based on fruits and vegetables, low-fat dairy products and whole grains demonstrated inverse relationships with risks of NAFLD^(29,30)^ as well as inverse associations in chronic diseases associated with NAFLD such as CVDs and diabetes^(31)^. Furthermore, coffee has been reported as an antioxidant-rich nutrient for the risk reduction of NAFLD. Recently, a meta-analysis has suggested that coffee intake in a dose-dependent manner decreases risks of developing NAFLD^(32)^. The ability of other antioxidant-rich nutrients to increase plasma TAC and hence decrease risks of NAFLD through modifications of oxidative stress has been well documented. These nutrients include bayberry juice^(33)^, chocolate^(34)^, onion^(35)^, lettuce^(36)^ and tomato products^(37)^. Findings have been verified even in supplementary interventions with antioxidants. Furthermore, beneficial effects of the combination of antioxidant supplements have been reported^(38)^, as synergistic effects of simultaneous administration and combination of one or more antioxidant supplements decrease risks of NAFLD^(38)^.

Oxidative stress has been shown to involve in pathogenesis and development of lipid metabolism disorders and insulin resistance and can lead to NAFLD^(39)^. Decreases in antioxidant defence mechanisms can increase lipid peroxidation, damage cellular organs and enzymes, and cause insulin resistance^(40)^. Thus, high dietary antioxidants can improve lipid and glucose metabolism disorders and decrease risks of NAFLD by protecting liver cells^(9,16,40)^. Additionally, high intakes of antioxidant-rich nutrients from plant foods, as part of a healthy diets, may include health benefits not only by protecting cells from oxidative damages^(41)^ but also by providing fibres and antioxidant nutrients such as vitamin D, vitamin C, vitamin E and magnesium, which are advantageous for BMI, serum lipids and glucose levels^(42)^. Beneficial synergetic effects of antioxidants, fibres, potassium, magnesium and other phytochemicals on the prevention of NAFLD have been observed in various studies^(42,43)^. In contrast, overweight and abdominal obesity have been suggested as potential risk factors for the progression of NAFLD^(44)^.

Dietary antioxidants provide protective mechanisms against obesity linked disorders, including inhibition of fat absorption, promotion of catabolism in adipose tissues, inhibition of proliferation, differentiation and angiogenesis in preadipocytes and induction of apoptosis in mature adipocytes^(45)^. Regarding these mechanisms, the mean weight of patients significantly decreased within the tertiles of DTAC. However, the present study included limitations that might affect interpretation of the results. Similar to other case–control studies, causal relationships between DTAC and NAFLD were not investigated. Moreover, the use of 168-item FFQ questionnaires made participants tired and biases in their responses, which were resolved by a trained questioner. Furthermore, the lack of a more accurate tool to assess energy and nutrient intake and also conditions of foods such as growth, cultivation, storage, processing and cooking conditions and the assay methods might affect antioxidant contents of the foods and study present^(21)^. Finally, the lack of an important data on NAFLD stage (liver biopsy) was another limitation to our study. Despite these limitations, this was the first study to investigate relationships between DTAC and NAFLD using a case–control design. However, the effects of confounders were tried to eliminate as far as possible using adjustment of a wide range of variables and validated questionnaires.

## Conclusion

Findings have shown that high DTACs are associated with decreased odds of NAFLD in adults, suggesting that the promotion of naturally elevated antioxidant capacities may help prevent developments of NAFLD. Encouraging consumption of diets with high antioxidant capacities is important in nutritional interventions for the prevention of NAFLD. However, complementary studies are necessary to further investigate associations of DTAC intakes with risks of NAFLD in other settings.
